# What Drives Generation Z to Avoid Food Waste in China? An Empirical Investigation

**DOI:** 10.3390/foods14020323

**Published:** 2025-01-20

**Authors:** Xin Qi, Muyuan Li, Jiayi Chen, Guohua Zhan, Lu Niu

**Affiliations:** 1 Management College, Ocean University of China, Qingdao 266100, China; x.qi@ouc.edu.cn (X.Q.); limuyuan@stu.ouc.edu.cn (M.L.); 22110031002@stu.ouc.edu.cn (J.C.); zghzhanguohua@163.com (G.Z.); 2 Institute of Marine Development, Ocean University of China, Qingdao 266100, China; 3 Innovation and Entrepreneurship Research Center, Ocean University of China, Qingdao 266100, China

**Keywords:** avoid, food waste, Generation Z, scarcity mindset, TPB

## Abstract

Avoiding food waste has become an important global issue. Given the global impact of food waste and the profound influence of Generation Z on future development, it is crucial to guide them in cultivating awareness and behaviors to reduce food waste, thereby promoting sustainable development. Considering young consumers’ specific characteristics and consumption environment, this study extended the Theory of Planned Behavior (TPB) framework by adding two constructs of moral self-identity and scarcity mindset. An online survey was conducted, receiving 417 valid responses, and the data were analyzed using structural equation modeling. This study shows that subjective norms, attitudes, and perceived behavioral control positively influence Generation Z’s intentions to avoid food waste. Meanwhile, moral self-identity remarkably positively influences attitudes and perceived behavioral control, which in turn affects intention to avoid food waste. Moreover, the positive moderating role of scarcity mindset is verified. This study refines the exploration of food waste within the realm of the Generation Z group, and the findings are beneficial for relevant stakeholders to further develop personalized promotion strategies for Generation Z.

## 1. Introduction

Food waste has become a critical global issue, with far-reaching implications for resource depletion and environmental pollution [1,2]. The United Nations Environment Programme (UNEP) and WRAP’s 2021 Food Waste Index Report highlight that approximately 17% of food available to consumers worldwide is wasted [3]. This phenomenon not only reflects an enormous waste of resources, but also underscores the critical issue of global food insecurity. According to the 2023 Global Food Crisis Report, over 250 million people faced severe hunger in 2022, with seven countries at risk of famine [4]. Furthermore, UNEP’s 2024 report points out that more than one billion tons of food are wasted annually, while 783 million people continue to suffer from hunger [5]. This stark contrast between waste and need drives home the urgent need for sustained efforts to combat food waste and its impact on global sustainability [6].

As the world’s largest developing country, China faces rising economic growth alongside growing food production [7]. However, food waste remains a significant issue that cannot be overlooked [8]. According to the 2023 China Food and Nutrition Development Report, China’s food loss and waste rate stands at 22.7%, with an economic loss of RMB 1.88 trillion in 2022, accounting for nearly 22.3% of the country’s agricultural output [9]. Notably, food waste behaviors are particularly pronounced in urban areas, where rising incomes, large portion sizes, and evolving consumption habits contribute to significant waste. In fact, approximately 9.4% of food waste in China occurs at the consumption level, making it the largest contributor compared to food production or retail waste [9]. The expansion of the middle class and urban population has driven higher consumption, leading to challenges in managing food waste effectively. To mitigate global food shortages and reduce environmental impact, China must focus on addressing waste at the consumer level, offering valuable insights for other nations striving to meet sustainable development goals. Addressing food waste behavior at the consumer level, particularly among young, urban populations, could serve as a critical strategy for curbing waste and promoting sustainability in China.

Generation Z (born between 1995 and 2009) is rapidly becoming a key consumer group, and their behaviors related to food waste are crucial for both global and Chinese sustainability efforts [10]. Compared to previous generations, Generation Z is more socially responsible and environmentally conscious, often choosing brands that emphasize sustainability and ethical practices [11,12]. According to the 2023 China Food and Nutrition Development Report, a significant portion of food waste in China occurs at the consumption level, and Generation Z is the primary demographic group involved in this behavior [9]. With China’s rapid urbanization and rising income levels, the consumption behaviors of Generation Z are expected to have a profound impact on national strategies to reduce food waste and promote sustainability. Recent studies show that younger generations, particularly Generation Z, are increasingly focused on sustainability and the principles of the circular economy. This growing concern has been seen in multiple sectors, with Generation Z demonstrating a preference for eco-friendly products and practices, making them more likely to support brands that prioritize environmental sustainability. These emerging trends reflect the increasing environmental awareness that drives Generation Z’s attitudes and behaviors in both food waste and consumption patterns [13,14]. Existing studies have paid limited attention to the food waste behavior of Generation Z. Only a few studies [15,16] have explored their food waste behaviors, and there is still a lack of sufficient in-depth research on the underlying mechanisms and interventions that influence the food waste behaviors of Generation Z. This research gap is particularly prominent in China. Research on effective strategies to prevent food waste is of significant importance. Not only will it help reduce resource waste and alleviate environmental pressure, but it will also contribute to achieving sustainable development goals [17]. By exploring the psychological mechanisms, attitudes, and intentional behaviors of Generation Z that prevent food waste, this research can provide a theoretical basis for more targeted policies and social interventions, ultimately resulting in long-term social and ecological benefits. Therefore, there is an urgent need for research to understand how to prevent food waste behavioral intentions among Generation Z.

Various theories can be applied to explain individual behavior. Among these, the Theory of Planned Behavior (TPB), introduced by Ajzen [18], has been widely and successfully applied in food consumption studies [19,20,21]. However, the TPB has faced criticism for several reasons, particularly regarding its insufficient consideration of other important variables. It has been suggested that these variables evolve and adapt in different contexts and behavioral scenarios [22]. To better understand Generation Z’s behavior of avoiding food waste, it is crucial to consider potential factors that could play an interesting role. Given the complexity of the ethical issues surrounding the problem of food waste [23], there is a clear need for more in-depth exploration. In addition, the impact of scarcity cannot be ignored, whether in the form of the actual availability of food resources in certain regions or in the sense of scarcity created by factors such as population growth and changing consumption patterns. Scarcity fundamentally changes the way people view food waste. It instills a sense of urgency and value and makes people more aware of the consequences of their actions [24,25]. Moral self-identity, reflecting a strong sense of responsibility, is particularly prominent among Generation Z, who are more socially conscious and environmentally aware [26]. This is because they are more exposed to social and environmental education in school and through various media platforms, which has cultivated their sense of moral obligation. They are more likely to internalize moral values related to sustainability. This identity could influence their attitudes and behaviors toward food waste [27,28]. Meanwhile, scarcity mindset, which is the belief in the limited availability of resources, also plays a crucial role for Generation Z. Growing up in an era of digitalization and resource scarcity, Generation Z tends to be more concerned about resource sustainability [29,30]. Therefore, this study integrates these two factors into the TPB framework to better understand Generation Z’s behavior of avoiding food waste and inform targeted interventions to promote sustainable consumption.

This study aims to construct a suitable framework to explain the key factors influencing Generation Z consumers’ intentions to avoid food waste. To achieve this, the study is based on the TPB, extracting variables from the TPB model (i.e., intention, attitude, subjective norm, perceived behavioral control), with a special consideration of moral self-identity and scarcity mindset, to build an extended framework. This study conducts a fit analysis to verify the applicability of the extended model in the context of food waste among Generation Z in China and uses path analysis and moderation tests to examine the implementation of each construct. This research fills the gap in the study of food waste prevention among Generation Z, a specific group. The findings provide valuable insights for psychological interventions to reduce food waste behaviors in Generation Z, contributing to the development of food waste prevention strategies. Moreover, the significance of this study also lies in providing empirical support for social and policy-level interventions, helping policymakers to design more effective public education approaches and interventions to encourage Generation Z to adopt sustainable consumption habits. By gaining a deeper understanding of the psychological motivations and behavior patterns of Generation Z, this research offers valuable insights for the food industry and environmental organizations, contributing to the global goals of promoting green consumption and sustainable development.

## 2. Theoretical Framework and Development of Hypotheses

### 2.1. Development of Hypotheses

#### 2.1.1. Variables from the TPB Model

The Theory of Planned Behavior (TPB) posits that behavioral intention directly influences individual behavior, and that the three elements of attitude, subjective norms, and perceived behavioral control (PBC) collectively affect behavioral intention [18]. As a well-established social psychological theory, the TPB effectively explains rational behaviors that are not fully controlled by individual will, and has broad applicability in the field of individual behavior research [31,32,33,34]. The strengths of the TPB include the following: identifying perceptions of the behavior under study and continuously assessing the importance of its components; being considered as one of the most effective models for behavior intervention; and offering a flexible structure, where additional variables can be added to extend the theory [31]. In food waste research, scholars have utilized the TPB to explore behavior, incorporating contextual factors like motivation and financial attitudes [35], or integrating it with other behavioral theories for a comprehensive approach [36].

- Attitude

Attitude refers to a persistent predisposition of liking or disliking that an individual holds toward a particular object or behavior. It can also be understood as the positive or negative feelings or evaluations that an individual has about a specific behavior. According to the Theory of Planned Behavior (TPB), a person’s attitude toward a particular behavior influences their behavioral intentions. It has also been suggested that attitude is often the most powerful predictor of behavioral intention [37]. Generation Z, known for their strong environmental awareness [38], tends to embrace and practice a zero-waste lifestyle [39], which suggests that they hold a more positive attitude toward reducing food waste. The intention to avoid food waste is largely influenced by Generation Z’s attitude toward it. When Generation Z holds a more positive attitude toward avoiding food waste, their intention to do so becomes stronger. Thus, the following hypothesis is proposed in this study:

**Hypothesis 1 (H1):**  *Attitude has a significant positive effect on intention to avoid food waste.*

- Subjective Norms

Subjective norms refer to the perceived pressure from significant others to support or oppose a behavior, shaped by normative beliefs and motivations to conform. Preferences of family [40], peers [41], and social media influencers [42] can subtly guide consumer decisions. For example, in China, family members often emphasize cultural values like frugality and respect for food, encouraging reductions in food waste. Similarly, peers and influencers on platforms like Weibo and Douyin (TikTok) have become key sources of normative influence. Previous research highlights the significant role of subjective norms in shaping behavioral intentions, such as promoting eco-friendly purchases [43], though some studies suggest their impact on green behaviors may vary [44]. In China, Generation Z, influenced by campaigns like “empty plate,” experiences greater pressure to adopt sustainable behaviors, strengthening their intention to avoid food waste. Based on this, the following hypothesis is proposed in this study:

**Hypothesis 2 (H2):**  *Subjective norms have a significant positive effect on intention to avoid food waste.*

- Perceived Behavioral Control (PBC)

Perceived behavioral control (PBC) refers to an individual’s perception of the ease or difficulty in performing a behavior, influenced by control beliefs and their strength. Unlike attitude and subjective norms, PBC reflects objective factors such as experience, time, and available resources. For Generation Z, elements like food storage facilities, convenience of food takeout services, and time management skills influence their PBC. For instance, limited storage space or busy schedules can hinder their ability to avoid food waste. When individuals perceive fewer obstacles and more resources, their confidence in reducing food waste increases, thus enhancing their intention to do so [45]. Previous research, such as Jia Li et al.’s study on college students, has identified PBC as a key determinant of food waste reduction intentions [46]. Accordingly, the following hypothesis is proposed in this study:

**Hypothesis 3 (H3):**  *PBC has a significant positive effect on intention to avoid food waste.*

#### 2.1.2. Incorporating Additional Constructs in the TPB

- Moral Self-identity

Moral self-identity refers to an individual’s recognition of their moral qualities, reflecting how central moral values are to their self-concept [26]. Those with a strong moral self-identity feel a responsibility to act in alignment with their values [47], making them more likely to view reducing food waste as a personal moral responsibility [48]. They experience guilt and responsibility toward wasteful behaviors, which motivates them to adopt positive attitudes and enhances their perceived behavioral control (PBC) in food waste avoidance [49]. Conversely, weaker moral self-identity may result in less motivation to act responsibly. This concept has been widely recognized as a reliable predictor of pro-social behavior [50], influencing both intentions and actions [51]. Additionally, individuals with a strong moral self-identity prioritize their internal moral code over external influences like convenience, which drives them to reject wasteful behaviors and feel they have the responsibility and ability to make the right choices [52,53]. This moral self-discipline enhances PBC, reinforcing their motivation to act in ways consistent with their values [54]. Based on this, the following hypotheses are proposed in this study:

**Hypothesis 4 (H4):**  *Moral self-identity has a significant positive effect on attitude.*

**Hypothesis 5 (H5):**  *Moral self-identity has a significant positive effect on PBC.*

- Scarcity Mindset

In traditional economics, resource scarcity refers to objective actual scarcity, but Mullainathan and others suggest it is a relative concept, where individuals feel they have fewer resources than needed for survival and development, which is known as the scarcity mindset [29]. Previous studies offer various definitions of the scarcity mindset. For instance, Hamilton et al. (2019) describe it as a state where an individual’s ability to meet their needs is threatened due to limited resources [55]. Tripathi et al. define it as a perception of having a fixed and finite amount of resources to meet demands [56]. In the context of food waste avoidance, this study adopts the definition by Mehta et al., where individuals perceive that the supply of resources is insufficient to meet their normal growth needs [57]. The scarcity mindset significantly influences individuals’ affective, cognitive, and behavioral styles. Research shows that a scarcity mindset leads to valuing behaviors, compensatory behaviors, and convergent behaviors [58]. Specifically, individuals with a scarcity mindset are more likely to increase their effective utilization of resources. Gao et al. demonstrated that activating the scarcity mindset can reduce food waste behavior [59]. Based on this, the following hypotheses are proposed in this study:

**Hypothesis 6 (H6):**  *Scarcity mindset plays a positive moderating role in the influence of attitudes* (H6a)*, subjective norms* (H6b)*, and PBC* (H6c) *on the intention to avoid food waste.*

### 2.2. Theoretical Framework

**Figure 1:**
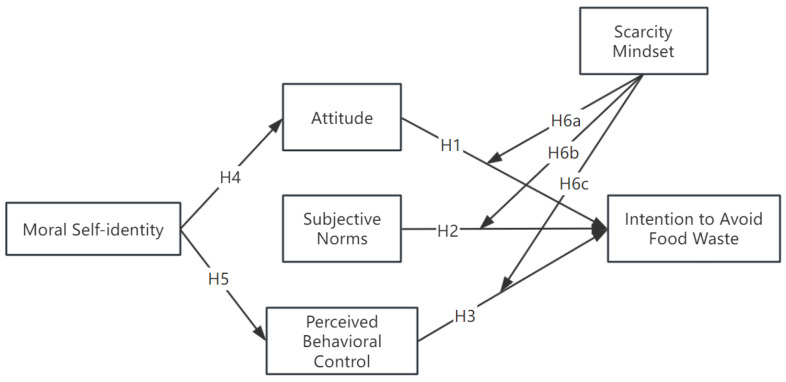
Conceptual model.

Based on the TPB model, this study proposes a new framework by adding two variables: moral self-identity and scarcity mindset. To better present the hypotheses proposed in this study, the research structure is illustrated in Figure 1.

## 3. Materials and Methods

### 3.1. Data Collection

This study collected data to analyze the proposed conceptual framework through an online questionnaire posted on a professional survey platform (www.wenjuan.com). Recognizing that living habits may vary across regions, which could lead to differences within Generation Z, the survey was conducted in China, utilizing online platforms and social media channels popular among Generation Z. This approach helps mitigate the potential bias caused by geographical factors, ensuring the sample’s representativeness and universality. Before the official launch, a pre-test was conducted with 30 randomly selected young consumers to refine the questionnaire and improve its clarity. This study specifically targeted Chinese Generation Z, and thus, an age selection question was included at the beginning of the survey to ensure only participants meeting the age criterion could proceed. In total, 470 questionnaires were collected, and 53 invalid responses were removed after data cleaning and logical consistency checks, leaving 417 valid responses (a response rate of 88.7%). According to Kline’s recommendation [60], at least 10 valid responses per parameter are required for valid empirical analysis. With 25 measurement items, the minimum sample size needed was 250 usable responses, making the 417 valid responses sufficient for the subsequent data analysis.

### 3.2. Measures

This study is based on reliable previous research scales and selected measurement items from them. The content of the scale refers to established Chinese–English bilingual scales to ensure the accuracy of the expression to the content of the measurement items. The questionnaire includes two parts. The first part is questions on attitude, subjective norms, PBC, moral self-identity, and scarcity mindset. Items were measured using a five-point Likert-scale approach, divided into five levels (level 1 for strongly disagree and level 5 for strongly agree) for measurement. The specific items of the questionnaire and the sources used for them are shown in Table 1. The second part counted the demographic characteristics of the respondents to the questionnaire with five items.

### 3.3. Data Analysis

In this study, the data were analyzed using Analysis of Moment Structures (AMOS) version 29 and Statistical Package for the Social Sciences (SPSS) version 29. Initially, we applied SPSS for descriptive analysis to observe the characteristics of the participants. Then, we used AMOS to conduct structural equation modeling (SEM) analysis in two steps. Firstly, validated factor analysis (CFA) was used to test the reliability and validity of the measurement model. Secondly, *p*-value, t-value, and standardized regression coefficient (β) were used to measure the complete structural model. Thus, model fit effects and hypothesized relationships could be assessed.

## 4. Results

### 4.1. Profile of the Respondents

The demographic characteristics of the respondents are presented in Table 2. The platform data indicated that the respondents came from 29 different provinces and regions across China, with the largest numbers originating from Shandong, Guangdong, and Henan. In terms of gender distribution, 67.8% of the respondents were female and 32.2% were male. The majority of participants were over 20 years old and had a high level of education. Specifically, 32.2% had completed high school or vocational education, while 64.1% held advanced degrees, including bachelor’s or master’s degrees. The level of monthly disposable income was relatively consistent across different subregions.

### 4.2. Measurement Model: Reliability and Validity

Table 3 presents the reliability and validity analysis of each measure. The results of the analysis showed that the Cronbach’s coefficients of intention to avoid food waste, subjective norms, attitudes, PBC, moral self-identity, and scarcity mindset were 0.785, 0.809, 0.783, 0.786, 0.794, and 0.823. Cronbach’s coefficients of the whole questionnaire reached above 0.7, which indicated that the data of the sample had good reliability. The KMO test assessment value (0.91 > 0.5) showed the adequacy of the sample taken, and Bartlett’s spherical test determination (*p* < 0.05) confirmed the statistical significance of the correlation matrix between the variables, indicating that the data collected were suitable for use in conducting factor analysis. The results of the confirmatory factor analysis showed that the loadings of the items ranged from 0.714 to 0.804; these results were better than the 5% significance level and the benchmark of 0.4 for a sample size of 200 or more. The test items all had significant unidimensionality and good model fit. Table 4 demonstrates the square root of AVE for each construct, all of which are greater than the Pearson correlation coefficient values for each latent variable, indicating that the discriminant validity of the data is good.

### 4.3. Structural Model: Goodness-of-Fit Statistics

Table 5 shows the goodness-of-fit indicators of the structural model. The data analysis shows that the theoretical framework proposed by the study has a good model fit (X^2^/df = 1.281; RMSEA = 0.026; RMR = 0.042; GFI = 0.95; NFI = 0.95; CFI = 0.985) and all the indices meet the criteria. The model is acceptable and has a satisfied ability to explain behavioral intentions.

### 4.4. Hypothesis Testing

Table 6 tests and demonstrates the proposed substantive impacts of the hypothetical paths. The results showed that attitudes (β = 0.248, t = 3.703, *p* < 0.001), subjective norms (β = 0.382, t = 5.640, *p* < 0.001), and PBC (β = 0.197, t = 3.095, *p* < 0.01) towards Generation Z avoiding food waste in China significantly and positively influenced the extended TPB modeling on the intention to avoid food waste, as supported by H1 and H3. The moral self-identification of Generation Z significantly and positively affects attitudes and PBC (β = 0.681, t = 9.517, *p* < 0.001; β = 0.596, t = 8.532, *p* < 0.001), as suggested by H4 and H5; thus, H4 and H5 are supported.

Hypothesis H6 of this paper proposes that scarcity mindset plays a positive moderating role in the relationship between attitudes (H6a), subjective norms (H6b), PBC (H6c), and the intention to avoid food waste, i.e., scarcity mindset strengthens the effects of subjective norms, attitudes, and PBC on the intention to avoid food waste. In order to further test the hypothesis, this paper adopts a three-step test of hierarchical regression analysis and uses the interaction term of the variables to test the moderating effect of information cognition, which means that if ΔR^2^ is significant when the interaction term is added, then a moderating effect exists.

The specific results of the test are shown in Table 7, Table 8 and Table 9. The empirical results of Model 1 in Table 7 show that attitude has a significant positive effect on the intention to avoid food waste, which further validates hypothesis H1. Model 2 in Table 7 shows that both attitude and scarcity mindset have a positive effect on the intention to avoid food waste, with regression coefficients of 0.41 (*p* < 0.001) and 0.104 (*p* < 0.05), respectively. Meanwhile, the results of Model 3 in Table 7 show that the regression coefficient of the interaction term is 0.246 (*p* < 0.001) and ΔR^2^ = 0.063 (*p* < 0.001). Thus, scarcity mindset plays a positive moderating role in the effect of attitude on the intention to avoid food waste. Similarly, we analyzed the empirical results in Table 8 and Table 9, and all regression coefficients and ΔR^2^ results met the test requirements. So, Hypotheses H6a, H6b, and H6c are established.

## 5. Discussion

The issue of food waste has become increasingly critical in contemporary society, with urgent attention needed to address its consequences. In particular, Generation Z is emerging as a significant force driving social change, particularly in terms of consumption habits, environmental awareness, and social responsibility. This study explores the factors influencing the food waste avoidance intentions of Chinese Generation Z from a psychosocial perspective. Building on the TPB, two additional constructs—moral self-identity and scarcity mindset—were incorporated into the framework, offering a comprehensive explanation of the factors avoiding Generation Z’s food waste behavior.

Through questionnaire research and data analysis, we found that attitudes, subjective norms, and perceived behavioral control (PBC) derived from the TPB model had a positive and significant effect on Generation Z’s intentions to avoid food waste, supporting findings from previous studies [35,36,67]. Notably, subjective norms had a stronger influence than both attitudes and PBC. This aligns with the findings of Russell et al. [36], who also observed a significant role of social influence in shaping pro-environmental behaviors. One possible explanation for this is the unique socio-cultural context and group characteristics of Generation Z, which amplify the role of subjective norms. In the unique socio-cultural context of China, collective values and peer influence are deeply rooted in decision-making processes [68,69]. This is especially true for Generation Z, who grew up in a highly socialized and digitalized environment and are therefore more easily driven by social influences and external expectations [70]. Online media and national campaigns, such as the “empty plate” initiative, serve as key channels reinforcing the social norms surrounding food waste avoidance, amplifying their influence on Generation Z’s behavioral intentions [71,72]. Additionally, Generation Z values group identity and belonging, driven by a stable material life and life stage characteristics that heighten their need for social connection, respect, and self-fulfillment. As a result, they are more inclined to conform to social behavioral norms [73,74]. In contrast, attitudes and PBC are more shaped by personal experience and internal beliefs, making their influence comparatively smaller. Attitudes are more shaped by personal beliefs and experiences, which may vary greatly among individuals. Previous research has shown that PBC is influenced by environmental constraints [75]. For Generation Z in China, many individuals reside in dormitories or shared apartments, where limited access to food storage and reuse facilities is common. This living situation reduces their perceived control over food waste, as practical constraints hinder their ability to implement waste-avoidance strategies effectively. However, it should be noted that although the influence of attitudes and PBC is relatively weaker, they still play an important role in the overall behavior intention formation process.

Meanwhile, the results of this study indicate that moral self-identity has a significant positive impact on attitudes and PBC, which is consistent with the results of previous human behavioral studies [52,53,71]. Therefore, relevant stakeholders, such as policymakers and educators, can consider promoting food waste avoidance by strengthening Generation Z’s moral self-identity. By reinforcing moral education and public awareness, various sectors of society can provide opportunities for Generation Z to engage in moral practices, thereby stimulating their sense of moral responsibility to reduce food waste. Additionally, businesses can build a positive brand image by actively participating in food waste reduction initiatives and advocating for environmental protection, which may help gain the trust and goodwill of Generation Z [72]. Furthermore, this study finds that scarcity mindset positively moderates the relationship between subjective norms, attitudes, PBC, and intention to avoid food waste [68]. This suggests that Generation Z’s inclination to avoid food waste may grow significantly in the future. As a result, governments and marketers should capitalize on this opportunity to enhance Generation Z’s awareness of conservation. Through targeted advertisements and promotional sales of food nearing expiration, marketers can raise awareness about the food waste issue, guide consumers in developing a food conservation mindset, and attract greater consumer attention and support.

## 6. Conclusions

This study explores the food waste avoidance behaviors of Chinese Generation Z, a key demographic shaping the future, through an extended TPB model. By integrating moral self-identity and scarcity mindset into the TPB framework, this research highlights the significant roles of attitude, subjective norms, PBC, moral self-identity, and scarcity mindset in influencing food waste avoidance intentions. The contributions of this study, both theoretical and practical, can be summarized in three key areas. First, the introduction of moral self-identity and scarcity mindset expands the TPB model and deepens our understanding of the psychological mechanisms underlying food waste avoidance among Chinese Generation Z. The findings underscore the growing importance of these variables, particularly in the post-pandemic era, where their positive impact on food-saving behaviors is especially pronounced. Second, our empirical analysis confirms the robustness of the TPB model, revealing complex relationships between the variables and demonstrating the significant moderating effects of moral self-identity and scarcity mindset. These findings not only validate the TPB model, but also highlight the importance of considering emerging social influences on Generation Z’s behavioral intentions in specific contexts.

In addition, policymakers and educators can play a key role in enhancing the moral self-identity of Generation Z by incorporating food waste awareness into moral education curricula and public events. For example, community service events such as food drives or educational seminars can encourage young people to engage in practices that reinforce the moral responsibility to reduce food waste. Businesses can also utilize ethical messages in their marketing campaigns to emphasize shared social and environmental responsibility. In addition, the moderating effect of the scarcity mentality suggests that raising awareness of resource limitations can increase the willingness of Generation Z to avoid food waste. Governments and organizations can design targeted interventions, such as food expiration awareness campaigns, to highlight the consequences of food waste and encourage behaviors such as purchasing discounted items that are nearing their expiration date. Grocery stores and food delivery platforms can also offer incentives for choosing imperfect or excess food, aligning individual behaviors with conservation goals. Finally, addressing practical barriers is critical, as PBC is often influenced by environmental constraints. For example, equipping dormitories and apartments with better food storage facilities, such as community refrigerators or portioned food sharing platforms, could allow Gen Z to manage food more effectively.

## 7. Limitations

This study has several limitations that warrant further exploration in future research.

First, it focused only on Generation Z’s intentions to avoid food waste, rather than their actual food waste avoidance behavior. Given the well-documented intention–behavior gap [76], this study’s findings may not fully reflect real-world practices. Future research should extend this model to examine actual food waste behaviors, using observational methods or longitudinal designs to better capture the relationship between intentions and actions.

Second, the study was conducted solely within the Chinese Generation Z population. Due to China’s unique socio-cultural conditions, including the prevalence of only-child families and the resulting heightened expectations on this generation [77], the findings may have limited generalizability. Research has shown that subjective norms’ impact on behavior varies significantly across national contexts. Therefore, cross-national comparisons involving Generation Z from diverse cultural backgrounds could uncover important differences in food waste attitudes and behaviors, offering valuable insights for developing globally effective policies and interventions.

Third, the inclusion of a pathway from TPB constructs to moral self-identity (MSI) could complicate the model without a strong theoretical foundation, potentially diluting the study’s focus. While this pathway was not incorporated in the present study, we recognize its potential to provide valuable insights into behavioral mechanisms. Future research could consider exploring these interactions to further enrich the understanding of Generation Z’s food waste behaviors.

Fourth, this study relied on self-reported data, which introduces potential biases such as social desirability bias and recall errors. Future research could incorporate objective measures, such as food diaries or waste tracking technologies, to validate and supplement self-reported findings. These methods would enhance the scientific rigor of studies on food waste behaviors.

The insights gained from addressing these limitations can contribute significantly to the ongoing efforts to promote sustainable consumption and global food conservation.

## Figures and Tables

**Table 1:** Questionnaire items and their source of adoption.

Variable	Item	Measurement Item	Adopted From
Intention to Avoid Food Waste (IAFW)	IAFW1	I try to avoid wasting food in my life.	[46,61]
	IAFW2	To avoid food waste, I will be careful to order in moderation.	
	IAFW3	I’ll make a point of packing my leftovers.	
Subjective Norms (SN)	SN1	No one important to me is in favor of over-ordering.	[46,61,62]
	SN2	Many of my friends around me have responded to the “empty plate” campaign to reduce food waste.	
	SN3	Most people in my family would be careful to avoid wasting food.	
Attitude (AT)	AT1	There are still many hungry people in the world and it is immoral if we waste food.	[39,62,63]
	AT2	Avoiding food waste is a smart move.	
	AT3	It makes me sad when I see food being wasted.	
Perceived Behavioral Control (PBC)	PBC1	I think food waste can be avoided.	[61]
	PBC2	If I want to save food, I can always reach my goal.	
	PBC3	It’s not difficult to order as much as I eat when I eat out.	
Moral Self-identity (MS)	MS1	It makes me feel good to be a person who has qualities like these.	[64]
	MS2	I am actively engaged in activities that reflect these qualities.	
	MS3	It’s important to me to be a person who possesses these qualities.	
Scarcity Mindset (SM)	SM1	In our modern society with increasingly scarce resources, I tend to avoid food waste.	[65]
	SM2	I’ll try to avoid food waste due to fear of food shortages.	
	SM3	When my money is tight, I tend to try to avoid food waste as much as possible.	

**Table 2:** Demographic characteristics of the sample (*n* = 417).

Demographic	Variables	Frequency	Percent (%)
Gender	Female	283	67.8
	Male	134	32.2
Age	15–19	50	11.9
	20–24	194	46.6
	25–29	173	41.5
Education	Elementary school and below	3	0.7
	Junior high school	13	3.0
	Senior high school	84	20.2
	Technical secondary school	50	12.0
	University	239	57.3
	Master’s or above	28	6.8
Monthly Disposable Income	<RMB 2000	109	26.1
	RMB 2000–3000	65	15.7
	RMB 3001–5000	77	18.5
	RMB 5001–8000	78	18.8
	RMB 8001–10,000	52	12.4
	>RMB 10,000	35	8.5

**Table 3:** Measurement model: reliability and validity.

Constructs	Factor Loadings	Cronbach’s α	AVE	CR
IAFW1	0.737	0.785	0.551	0.786
IAFW2	0.768			
IAFW3	0.721			
SN1	0.74	0.809	0.588	0.810
SN2	0.764			
SN3	0.792			
AT1	0.754	0.783	0.546	0.783
AT2	0.732			
AT3	0.733			
PBC1	0.745	0.786	0.552	0.787
PBC2	0.717			
PBC3	0.765			
MS1	0.714	0.794	0.564	0.795
MS2	0.767			
MS3	0.77			
SM1	0.751	0.823	0.608	0.823
SM2	0.804			
SM3	0.784			

Note. IAFW: intention to avoid food waste; SN: subjective norms; AT: attitude; PBC: perceived behavioral control; MS: moral self-identity; SM: scarcity mindset; AVE = Average Variance Extracted (AVE = ∑SMC/(∑SMC + ∑standard measurement error); SMC = Squared Multiple Correlation (i.e., squared value of correlation between the constructs); CR = Composite Reliability.

**Table 4:** Correlation matrix for discriminant validity.

	INT	N	ATT	PBC	SE	SRM
IAFW	0.742					
SN	0.487 ***	0.767				
AT	0.444 ***	0.499 ***	0.739			
PBC	0.395 ***	0.411 ***	0.452 ***	0.743		
MS	0.34 ***	0.433 **	0.460 ***	0.396 **	0.751	
SM	0.216 ***	0.244 ***	0.253 ***	0.230 ***	0.209 ***	0.78

Note. *** and **, respectively, represent 0.001 and 0.01 levels of significance. Diagonal elements show square roots of AVE; off-diagonal elements represent correlations between constructs; IAFW: intention to avoid food waste; SN: subjective norms; AT: attitude; PBC: perceived behavioral control; MS: moral self-identity; SM: scarcity mindset.

**Table 5:** Summary of fit indices from confirmatory factor analysis.

Fit Indices	Model	Recommended Value	Results
X^2^/df	1.281	>1 and <5 *	Satisfactory
RMSEA	0.026	≤0.08 *	Satisfactory
RMR	0.042	≤0.05 *	Satisfactory
GFI	0.95	≥0.9 *	Satisfactory
NFI	0.95	≥0.9 *	Satisfactory
CFI	0.985	≥0.9 *	Satisfactory

Note. * Source: Bagozzi and Yi [66]; RMSEA: Root Mean Square Error Approximation; RMR: Root Mean Square Residual; GFI: Goodness-of-Fit Index; NFI: Normative Fit Index; CFI: Comparative Fit Index.

**Table 6:** Hypotheses test results.

Hypothesized Path	Standardized Path Coefficients	t-Value	Result
H1: AT→IAFW	0.248	3.703 ***	Support
H2: SN→IAFW	0.382	5.640 ***	Support
H3: PBC→IAFW	0.197	3.095 **	Support
H4: MS→AT	0.681	9.517 ***	Support
H5: MS→PBC	0.596	8.532 ***	Support

Note. IAFW: intention to avoid food waste; SN: subjective norms; AT: attitude; PBC: perceived behavioral control; MS: moral self-identity; ** *p* < 0.01; *** *p* < 0.001.

**Table 7:** H6a test results.

Variables		IAFW		
		Model 1	Model 2	Model 3
Constant Variables	Constant Term	2.222 ***	1.888 ***	1.017 ***
	Gender	0.08	0.076	0.056
	Age	0.137	0.133	0.105
	Education	−0.094 *	−0.089 *	−0.083 *
	Career	−0.067	−0.06	−0.043
	Monthly Disposable Income	0.043	0.04	0.044
Independent Variables	AT	0.436 ***	0.41 ***	0.475 ***
Moderator Variables	SM		0.104 *	0.241 ***
Interaction Terms	AT × SM			0.246 ***
	R^2^	0.215	0.225	0.288
	ΔR^2^	0.215 ***	0.01 *	0.063 ***

Note. * *p* < 0.05; *** *p* < 0.001; IAFW: intention to avoid food waste; AT: attitude; SM: scarcity mindset.

**Table 8:** H6b test results.

Variables		IAFW		
		Model 1	Model 2	Model 3
Constant Variables	Constant Term	2.247 ***	1.923 ***	1.113 ***
	Gender	0.067	0.064	0.038
	Age	0.106	0.104	0.112
	Education	−0.122 **	−0.115 **	−0.116 **
	Career	−0.019	−0.015	−0.021
	Monthly Disposable Income	0.021	0.02	0.032
Independent Variables	SN	0.466 ***	0.444 ***	0.547 ***
Moderator Variables	SM		0.097 *	0.210 ***
Interaction Terms	SN × SM			0.321 ***
	R^2^	0.257	0.265	0.345
	ΔR^2^	0.257 ***	0.009 *	0.080 ***

Note. * *p* < 0.05; ** *p* < 0.01; *** *p* < 0.001; IAFW: intention to avoid food waste; SN: subjective norms; SM: scarcity mindset.

**Table 9:** H6c test results.

Variables		IAFW		
		Model 1	Model 2	Model 3
Constant Variables	Constant Term	2.377 ***	1.953 ***	1.410 ***
	Gender	−0.016	−0.014	0.023
	Age	0.077	0.076	0.074
	Education	−0.087	−0.082	−0.084
	Career	−0.044	−0.037	−0.045
	Monthly Disposable Income	0.047	0.044	0.041
Independent Variables	PBC	0.418 ***	0.387 ***	0.449 ***
Moderator Variables	SM		0.130 **	0.188 ***
Interaction Terms	PBC × SM			0.227 ***
	R^2^	0.168	0.183	0.225
	ΔR^2^	0.168 ***	0.015 **	0.041 ***

Note. ** *p* < 0.01; *** *p* < 0.001; IAFW: intention to avoid food waste; PBC: perceived behavioral control; SM: scarcity mindset.

## Data Availability

The raw data supporting the conclusions of this article will be made available by the authors on request.
